# Retraction: Implementation of Learning Management Systems (LMS) in higher education systems through bipolar complex hesitant fuzzy Aczel-Alsina power aggregation operators: A case review for China

**DOI:** 10.1371/journal.pone.0352806

**Published:** 2026-07-08

**Authors:** The PLOS One Editors

The *PLOS One* Editors retract this article [1] due to concerns about compromised peer review.

In addition, the article cited as Reference 20 was retracted before publication of [1], and the articles cited as References 34 and 35 were retracted after publication of [1].

All authors did not agree with the retraction.
